# Understanding an Environmental Health Risk: Investigating Asthma Risk Perception in Ontario Youth Sport

**DOI:** 10.3390/ijerph16112033

**Published:** 2019-06-07

**Authors:** Francesca S. Cardwell, Susan J. Elliott

**Affiliations:** Department of Geography & Environmental Management, University of Waterloo, Waterloo, ON N2L 3G1, Canada; elliotts@uwaterloo.ca

**Keywords:** asthma, environment and health, risk perception, child and youth health, sport participation

## Abstract

The environment, broadly defined, plays a significant role in shaping human health. Understanding how environmental health risks are perceived by different people, in different places, and at different times is critical to their management. Using a place-based conceptual framework, this research investigates asthma risk perception determinants and outcomes amongst organized team sport stakeholders in Ontario. Two online surveys (coaches, n = 94; parents of athletes diagnosed with allergic disease, n = 90) were conducted. Binary regression was used to investigate determinants of risk perception. Asthma ranked seventh of 17 health hazards by coaches (23% ranked as high) and parents (34%), and determinants of risk included trigger knowledge, risk exposure, propensity for risk, indicators of trust, and socioeconomic variables (e.g., gender). As policy-makers look to manage health risks in sport, considering the risk profiles of different stakeholders (e.g., coaches, parents of vulnerable athletes), as well as the characteristics of the places in which risk is experienced, is critical to improving environment and health management in organized youth team sports.

## 1. Introduction

The environment plays a pivotal role in shaping human health. While healthy environments can prevent premature death and disease [1], a host of literature (e.g., The *Lancet’s* recent commission on pollution and health [2]) documents the health burdens that accompany the effects of environmental change. Managing health hazards associated with environmental factors can be especially challenging; in some contexts, there may be less certainty regarding the causes, exposures, and impacts (e.g., the social, demographic and economic disruptions of climate change [3]), challenging our ability to objectively quantify and manage the spatial and temporal magnitude of risk [4]. In this context, understanding how environmental health risks are perceived and managed not only by policy- and decision-makers, but by the public, is therefore particularly important. While risk management decision-makers are in a unique position to respond to environmental health risks, it is often with some level of uncertainty (e.g., through the use of the Precautionary Principle [5]). Further, risks are both real and socially constructed [6], and the general public often rely on personal knowledge and subjective experiences to define risk [4,7]; for these reasons, policy-makers must react to risks both perceived to be important by the general public [8,9], as well as those hazards objectively quantified as risky. In this way, understanding not only objective determinations of risk, but the way the public perceives and responds to risk is essential to improving risk communication and management.

### 1.1. Place-based Conceptual Framework

While traditional risk perception literature focuses on characteristics of specific hazards as determinants of risk perception (e.g., level of dread [7]), critiques have focused on the simplistic nature of these risk perception models. There has been increased recognition of the need for emphasis on the broader social, political, and cultural contexts in which risk is embedded, and the relationships with and between people, risk perception, and the places where risk is experienced [4].

**Figure 1:**
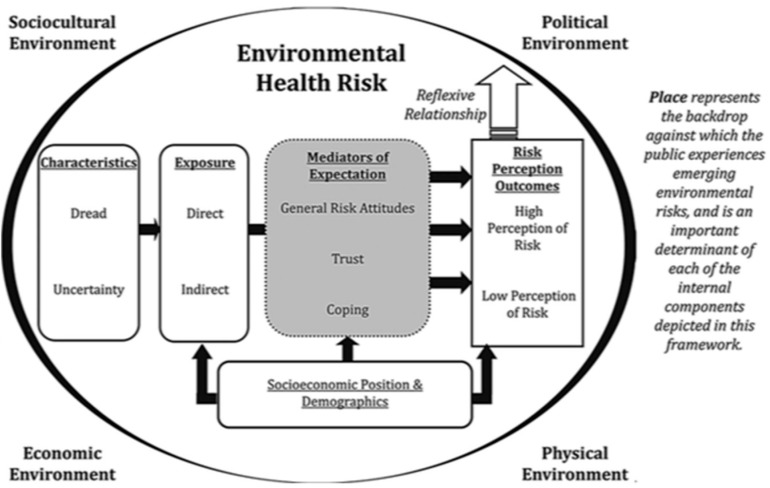
The conceptual framework for understanding emerging environmental risks [4].

To address these critiques, Harrington and Elliott [4] propose a framework to ensure the role of place is considered when investigating how publics perceive various environmental health risks. The framework builds upon three existing risk perception paradigms and the place effects on health literature; Slovic’s [10] psychometric paradigm, Douglas and Wildavsky’s [11] cultural theory paradigm of risk perception, and Kasperson et al.’s [12] social amplification of risk framework. Slovic’s [10] paradigm considers the individual demographic and cognitive characteristics that influence risk perceptions. More specifically, level of dread risk (e.g., the potential for negative consequences) and unknown risk (e.g., perceived level of control) of a specific hazard combine to shape risk perception. The cultural theory paradigm focuses on how a population’s social and cultural values and relationships contribute to the formation of different worldviews (e.g., hierarchical, fatalism), which in turn influence levels of perceived risk. The third paradigm, the social amplification of risk framework, posits that perceptions of risk can be amplified or attenuated based on the nature and relevance of the message being communicated and the channel of communication (e.g., through the mass media). Based on a lack of attention to the broad environmental contexts in the risk perception process, Harrington and Elliott specifically categorize the environment into the physical, economic, political, and sociocultural. Place therefore represents the backdrop of the framework, recognizing varying characteristics and meanings of space, and the role they play in shaping perceptions, behaviors, and health. The framework is depicted in Figure 1, and a detailed theoretical underpinning of the model is described at length elsewhere [4]. While the framework has been applied using food allergy risk perception data from a national Canadian survey [13], Harrington & Elliott (2015) call for future testing using different health risks, at different scales, using different methodological approaches, and with different populations. We aim to investigate asthma risk perception determinants and outcomes within coaches and parents in the organized youth team sport environment in Ontario, Canada.

### 1.2. Applying the Framework in the Context of Asthma

During the past few decades, asthma prevalence worldwide has increased, and asthma is now one of the most common chronic conditions affecting both adults and children [14,15,16]. Asthma is a chronic inflammatory disease of the airways and can present with a range of symptoms, including shortness of breath, wheezing, coughing, and chest tightness. Symptoms vary in severity and differ over time and on a case-by-case basis. While the complete etiology of asthma is unknown, we know that genetic predisposition and the environment play a large role as either protective or as risk factors (Subbarao et al., 2009). For example, factors that may influence asthma development include exposure to environmental tobacco smoke or air pollution [14], as well as lifestyle factors such as stress and physical inactivity [15]. A range of contributing factors are likely, and a leading theory that may partially account for the reported increase in allergic disease prevalence is the hygiene hypothesis. This theory suggests that although children exposed to Western lifestyles may be protected from traditional infectious disease burdens, exposure to protective microbes is necessary for healthy immune system response development. These children are therefore more susceptible to conditions related to hypersensitive immune systems, such as allergic disease [4,17]. While a genetic component exists, environmental factors play a significant role in the expression of allergic disease, including asthma. 

Though our understanding of asthma etiology is incomplete [15], asthma appears to be increasing in many parts of the world [14]. Asthma prevalence varies geographically and with socioeconomic status. For example, in Asian countries, prevalence (rates between 2–4%) appears to be lower than other Western countries including the United Kingdom, Canada, Australia, and New Zealand (where prevalence varies between 15–20%) [15]. Asthma is the most common respiratory disease in Canada [18]. In Ontario more specifically, the prevalence of asthma (15.4% [19]) increased by 70.5% from 1996–2005, likely due, in part, to the increasing incidence in children [20].

In addition to the physical symptoms of respiratory allergies and asthma, social and emotional impacts are reported, including decreased wellbeing and quality of life, feelings of difference, frustration, anxiety, and depression [21,22]. The psychosocial dimensions of living with asthma have other health-related impacts; for example, children with asthma are generally less physically active [16], for reasons including inaccurate symptom perception by caregivers or those diagnosed (e.g., perception that breathlessness or taking inhalers is harmful), individual or family illness beliefs (e.g., acceptance of physical inactivity as inevitable), or organization policies (e.g., limited asthma management knowledge in the school environment [23]) [16]. 

The impacts of asthma on children and youth are therefore diverse, and understanding how stakeholders in organized youth team sport perceive asthma risk is important for multiple reasons. Physically, as exercise increases ventilation in both speed and endurance athletes, exposure to potential asthma triggers (e.g., cold air, pollen) in different physical environments is enhanced among athletes [24,25]. Increased ventilation and exposure to pollutants close to the ground (e.g., ground-level ozone) [26], make child and youth athletes vulnerable to asthma development and worsening symptoms [25], and increases the risk associated with potential mismanagement (e.g., coaches encouraging athletes to compete through symptoms). Living with asthma also has emotional implications with respect to experiences in ‘safe’ risk environments [4,27]. For children and youth participating in organized sport as a form of physical activity, the management of environment and health risks is particularly unique. Not only do environment and health risks challenge those impacted (e.g., asthmatic athletes), but others who regularly interact with them; in organized sport, risk management can fall on caregivers, teammates, sports organizations, and coaches, many of whom are volunteers with little training in child development or environment and health issues [28]. Further, there is currently limited asthma education available for coaches [29], and inadequate prevention and management practices by those in charge could increase asthmatic youth athlete vulnerability. Parents and members of other social networks (e.g., teammates) are also involved in the risk-management process [30], and are therefore impacted by the physical and emotional experiences associated with living with and managing asthma in sport. 

The links between elements of the environment, asthma and its management, and the role of various stakeholders in organized youth team sports are inevitably complex. Increasing our understanding of how two groups (e.g., parents of athletes with allergic disease, coaches) within organized youth team sport perceive asthma is therefore critical to ensure children with asthma have the same opportunities for well-managed organized sport as their non-asthmatic peers. Applying Harrington & Elliott’s framework, we therefore investigate the broad factors that influence asthma risk perception outcomes in organized youth team sports in Ontario, Canada. This research therefore has two primary objectives: (1) To document asthma risk perception outcomes amongst youth team sports coaches and parents of allergic athletes, and (2) to investigate the factors shaping asthma risk perception amongst two distinct populations (coaches, parents) in youth team sport. 

## 2. Materials and Methods

The data stem from a larger mixed-methods study that seeks to understand how users and providers of sport in Ontario understand and manage the links between allergic disease, the environment and physical activity. Results from two online risk perception surveys will be reported. In addition to the quantitative surveys, we conducted qualitative semi-structured in-depth interviews with coaches, youth athletes and their parents, regarding their perceptions of asthma and its management in organized youth team sports. The online risk perception surveys were conducted between October 2013 and December 2014 in Ontario, Canada, and were granted ethics clearance by the University of Waterloo Research Ethics Committee. The project identification code from the University of Waterloo Office of Research Ethics is #19057.

The first survey was completed by community-level youth team sport coaches in Ontario. Both recreational and competitive, indoor and outdoor coaches were included (n = 94). The second survey was completed by parents of children diagnosed with allergic disease (n = 90). The surveys were hosted by FluidSurveys, which is now operated by SurveyMonkey (www.surveymonkey.com). Coach and parent participants were recruited through sports organizations in Ontario. A list of organized youth team sport organizations from the Greater Golden Horseshoe Region of Ontario (spanning municipalities including Guelph, Halton Region, Hamilton, Peel Region, Waterloo Region, and Wellington County) was compiled (N = 219 organizations) using an online search with key terms including a list of possible team sports (e.g., soccer, baseball, softball, rugby, cricket) and municipalities within the region. All clubs were contacted with a brief description of the research and asked to either post a link to the surveys on their website or distribute an email with the survey links to their membership. If coach email addresses were listed directly on an organization’s website, a summary of the research and invitation to participate was distributed. A research Twitter account was also created; sports organizations, and health, environment or asthma and allergy organizations (e.g., Asthma Canada) across Ontario and Canada were followed. Links to both surveys were periodically tweeted by the research account, which were then occasionally retweeted by followers. The coach and parent surveys were active from October 2013–September 2014, and November 2013–December 2014, respectively. For both surveys, a criterion for inclusion was being an Ontario resident. For coach participants, other inclusion criteria included coaching youth (under age 18) organized team sport within 12 months of participation, while parent participants were required to have a child aged 18 years or younger affected by asthma or allergic disease (e.g., food allergy, asthma, respiratory allergy), that participated in organized team sport within a year of survey completion. If criteria were not met, responses were removed from the dataset.

Harrington and Elliott’s [4] conceptual framework (Figure 1) and the relevant literature on asthma, physical activity, and sport participation were used to guide survey question design; questions cover a range of topics including the sport physical and sociocultural environments, risk exposure, indicators of levels of trust and general environmental risk attitudes, and risk perception outcomes. Socioeconomic position and demographic data were collected for both parent and coach samples (Table 1). While certain questions are consistent across the coach and parent surveys (e.g., environmental attitudes, symptom and trigger knowledge), some questions are unique to each survey (e.g., coaching qualifications, allergic child’s gender). 

The survey collected attitudinal data related to the environment and individual, family and Canadians’ health. These questions were used to measure concern about the possible impacts of the environment on human health, and used as an indicator of participant attitudes toward environmental health risks (as applied by Harrington et al. [13]). For efficiency, the five survey items were used to construct an Environment and Health Attitudes score (Cronbach alpha value of 0.810 and 0.739 for coaches and parents, respectively), whereby participants received a point for each statement in which they either ‘Agreed’ or ‘Strongly Agreed’ with a pro-environmental attitude (for a total of 5). Further, nineteen survey items on asthma symptoms were used to create an Asthma Symptom Knowledge score (Cronbach alpha value of 0.855 and 0.881 for coaches and parents, respectively), while twenty survey items on asthma triggers were used to create an Asthma Trigger Knowledge score (Cronbach alpha value of 0.905 and 0.891 for coaches and parents, respectively). These scores are used as indicators of participant knowledge of asthma. Cronbach’s alpha measure is used to assess the reliability of a set of scale items, and while there are differing reports about acceptable values of alpha, those between 0.7 and 0.95 are generally considered suitable.

Risk perception outcome data are measured for both parents and coaches, using Krewski et al. [31,32] and Harrington et al.’s [13] methodology as a guide. Coach and parent participants were asked to rate the degree of risk (e.g., “high”, “moderate”, “low” or “unknown”) posed to the Canadian population by asthma and 16 other health conditions. Risk ratings were grouped together to represent high risk, versus low risk (risks ranked as “moderate”, “low” or “unknown”). Respondents with no opinion were grouped into the unknown risk category. To increase understanding of whether respondents who rate health risks higher are more likely to rate asthma risk as higher, a Propensity for Risk score was calculated. For each of the 17 health risk variables, risks ranked as “high” were given 3 points, “moderate” were given 2 points, and “low” were given 1 point. The total possible Propensity for Risk score was 51.

Using the asthma risk perception measure (“high” vs. “other”) as the outcome variable, we conducted a bivariate analysis to investigate which variables of interest were correlated to asthma risk perception outcomes for the coach and parent surveys individually. Chi-square and Fisher’s exact tests (for categorical variables), and independent t-tests (for continuous variables) were conducted. In order to compare asthma risk perception outcome differences between coaches and parents, a Mann-Whitney U test was conducted.

Finally, binary logistic regression was used to investigate the determinants of asthma risk perception. Variables were chosen to be included in the coach and parent models based on significance in the bivariate analysis, and the relevant asthma and allergic disease risk perception literature. Overall fit for the models was assessed using the Omnibus Tests of Model Coefficients. All univariate, bivariate, and multivariate analyses were carried out using SPSS Statistics Standard Gradpack 25 for Mac (IBM, Armonk, NY, USA).

While Harrington & Elliott’s [4] framework was used to guide survey design, certain components of the framework (e.g., political and economic environments) were not directly addressed in our surveys. 

## 3. Results

Univariate results are presented in five categories drawn from Harrington and Elliott’s [4] place-based framework; Survey Sample Characteristics, Risk Perception Outcomes, Sociocultural Environment, Physical Environment, and Environmental Health Risk. Regression results are then detailed. 

### 3.1. Sample Characteristics

Compared with the Ontario population [33], coach and parent samples had higher proportions of younger and middle-aged respondents (46% of coach and 63% of parent respondents were between 40–49 years of age, compared with 14% of Ontarians). While coach participants’ gender is split fairly evenly (55% males), the large majority of parent respondents (77%) were female. Respondents with lower levels of education were under-represented, as 100% of coaches and parents had completed secondary school, compared with 82% of Ontarians. Coach and parent samples had a lower proportion of immigrants (14% and 19%, respectively), while a larger proportion of coaches (66%) and parents (81%) are married compared with Ontarians (58%) (Table 1). Although the surveys were open to participants from across Ontario, the Regional Municipalities of Halton and Waterloo, and Middlesex County have the largest proportion of respondents. 

### 3.2. Risk Perception Outcomes

**Figure 2:**
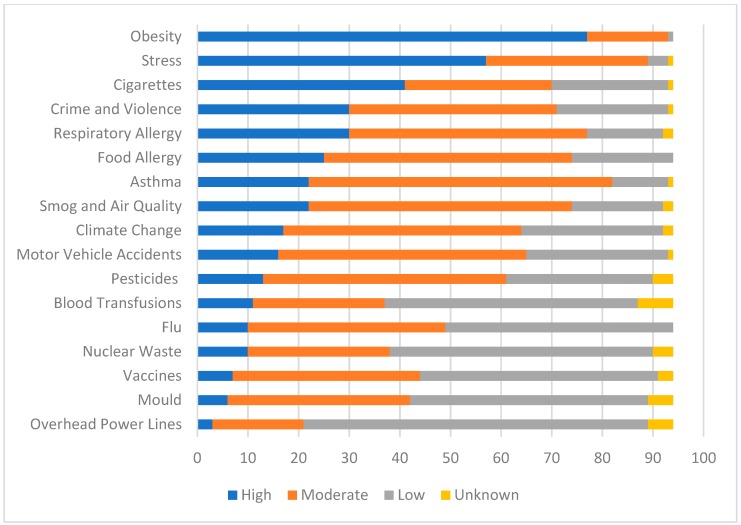
Coach Perceived Level of Risk to the Canadian Public for 17 Health Hazards.

**Figure 3:**
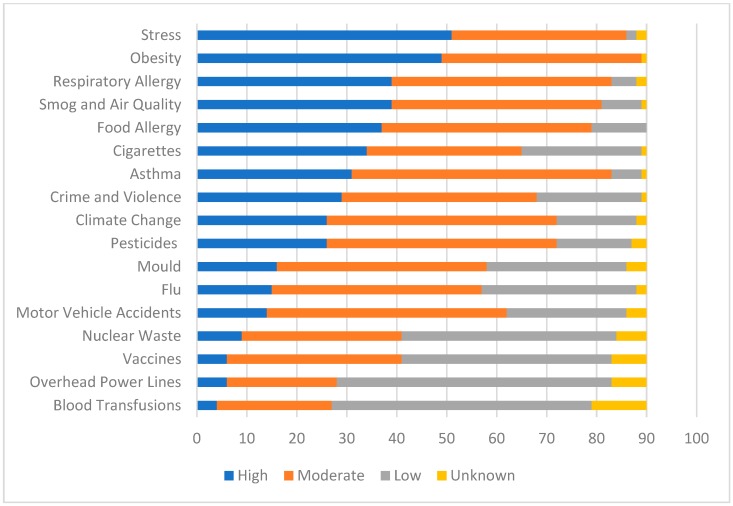
Parent perceived level of risk to the Canadian public for 17 health hazards.

To understand how coaches and parents perceive asthma risk compared with other environmental and health risks, participants were asked to rank seventeen health hazards as “high”, “moderate”, “low” or “unknown”. Figure 2 and Figure 3 illustrate the respective coach and parent perceived level of risk for the health hazards. Amongst coaches, obesity (82%), stress (61%), cigarettes (44%), crime and violence (32%), and respiratory allergy (32%) were the top five risks, while parents perceived stress (57%), obesity (54%), respiratory allergy (43%), smog and air quality (43%), and food allergy (41%) as their top risks. Asthma was ranked seventh by both coaches and parents; rated highly by 23% and 34%, respectively. Other allergic disease determinants and outcomes were also included as health risks, such as food allergy (ranked highly by 27% of coaches and 41% of parents), smog and air quality (23% of coaches, 43% of parents), and cigarettes (44% of coaches, 38% of parents). 

In order to determine whether asthma risk perception outcomes differ between coach and parent participants, a Mann-Whitney U test was conducted (risks ranked as “High” were scored as 3, “Moderate” as 2, and “Low” as 1). Distribution of the risk perception outcomes for coaches and parents were similar, as assessed by visual inspection. Parent mean risk perception scores were 2.26 (SD = 0.63), while coach mean risk perception scores were 2.10 (SD = 0.62). A Mann-Whitney U test showed that there was a significant difference (U = 3553.5, z = −1.812 *p* = 0.07) between coach and parent risk perception outcomes. 

### 3.3. Physical Environment

Based on the sports organizations contacted for recruitment, a range of physical environments, including outdoor grass and turf fields, outdoor baseball fields, and indoor ice rinks, pools, turf and gymnasium environments are relevant. More specifically, most coaches (59%) reported participating in their sport primarily in outdoor environments, while 33% coached solely in indoor environments. Parents also reported their allergic child’s primary sports environment; 12% participated solely in outdoor environments, 29% participated in indoor environments, while 59% were involved in both indoor and outdoor environments. Coach respondents also reported their season(s) of participation (Fall [76%], Winter [74%], Spring [80%], Summer [71%]), and typical training/game times (6 a.m.–9 a.m. [11%], 9 a.m.–12 p.m. [24%], 12 p.m.–4 p.m. [30%], 4 p.m.–6 p.m. [25%], 6 p.m.–9 p.m. [88%], 9 p.m.–12 a.m. [34%]).

### 3.4. Sociocultural Environment

Similarly, the surveys address the sociocultural context in which organized youth team sport occurs. As an indicator of the sociocultural environment, and to understand if coaches interact with parents of the athletes they coach related to athlete health, they were asked: “Do parents typically speak to you about their children’s health concerns?” Seventy-five percent reported discussing athlete health with parents. Similarly, 69% of parents (n = 62) state that they have discussed the symptoms of their child’s asthma/allergies with the coach. These 62 participants were asked if their coach was receptive, and all reported coach receptivity. More specifically, 57% of parents (n = 51) reported discussing the appropriate steps to manage their child’s asthma/allergies with the coach, and 100% of these parents described their coach as receptive. Of the 51 parents who reported discussing management with coaches, 82% (n = 42) identified that their coach followed through with the appropriate asthma management steps.

### 3.5. Environmental Health Risk

#### 3.5.1. Socioeconomic Position and Demographics

In addition to the sample characteristics, the coach and parent surveys investigate other general and sport-related demographics. Broadly, 71% of coaches reported having their own children (compared with 100% of the parent sample). When asked about the gender of their allergic child, parents most frequently (60%) reported having a female allergic child. More specifically related to sport participation, the majority of coaches (50%) fill head coach roles, while 19% and 31% of coaches fill assistant or other (e.g., goalkeeper coach) roles, respectively. Coaches were involved with a number of age groups (under 9 [14%], 10–13 [18%], 14+ years [35%], multiple age groups [32%]), and have a range of years of experience (0–5 [37%], 6–15 [45%], 16+ years [18%]). The majority of coaches reported some form of coaching (79%), or medical qualifications (e.g., First Aid) (65%). Coaches were involved frequently in competitive sport (54%) or with multiple teams in both competitive and recreational environments (18%). When asked about the level of their allergic child’s primary sport, the majority (62%) of parents also reported competitive sport. Coaches were primarily involved in soccer (56%), ice hockey (18%), and football (7%), while parents were involved most frequently with soccer (66%), ringette (20%), and ice hockey (14%).

#### 3.5.2. Risk Characteristics

While we did not directly investigate levels of dread risk, there is evidence to suggest asthma prevalence is increasing (Subbarao et al., 2009), and is increasingly common amongst children in Ontario (Gershon et al., 2010). Although asthma is involuntary and inequitable (e.g., disproportionately impacts children, minority groups), and has potentially fatal consequences (e.g., Ontario student Ryan Gibbons suffered a fatal asthma attack at school in 2012 [34]), it can be controlled (e.g., with medication) and has low widespread catastrophic potential. 

Amongst coaches and parents, asthma is relatively known. Amongst coaches, the median Symptom Knowledge score was 11 (mean = 10.37, SD = 3.67), and median Trigger Knowledge score was 14 (mean = 12.4, SD = 4.41). Amongst parents, the median for both Symptom Knowledge and Trigger Knowledge scores was 11 (Symptom score mean = 10.28, SD = 4.1; Trigger score mean = 10.72, SD = 4.05).

#### 3.5.3. Exposure

In total, 27% of coaches and 46% of parents reported a personal allergic disease diagnosis (direct exposure), and 27% of coaches identified having another family member (e.g., child, parent, sibling) affected by allergic disease (indirect exposure). While all parents have a child with allergic disease, 77% report an asthmatic child specifically, and 60% have a child with another form of allergy (e.g., food, other respiratory allergy). Also, 42% of parents reported a child affected by 3+ forms of allergic disease.

#### 3.5.4. Mediators of Expectation

Amongst coaches, the Propensity for Risk score varied between 18 and 45, with a median ranking of 32 (mean = 31.89, SD = 6.39). Amongst parents, the score varied between 18 and 47, with a median of 34 (mean = 33.29, SD = 6.53). 

Due to the role of the environment in the etiology of asthma, and in order to understand how general environmental attitudes mediate asthma risk perception, coach and parent participants were asked to rank their agreement with a series of five statements related to the environment and individual, family, and Canadians’ health. Coach participants generally expressed higher levels of concern related to the environment broadly (e.g., 60% reported being concerned about the effects of the environment on the health of their friends and family), but less related to questions about climate change and health (e.g., 34% reported concern related to the health impacts of climate change on family and friends). Seventy-nine percent of parent participants reported high levels of concern related to the effects of the environment on the health of their friends and family, and the majority (55%) expressed worry related to the health impacts of climate change on their family and friends. For efficiency, an Environmental Attitudes score was constructed. Amongst coaches, the Environmental Attitudes score varied between 0 and 5, with a median ranking of 3 (mean = 2.64, SD = 1.83). Amongst parents, the score varied between 0 and 5, with a median ranking of 3.5 (mean = 3.43, SD = 1.61).

Finally, measures of trust and coping were included in the surveys, as participants were asked to rank a series of statements related to the social environment and coping mechanisms on their team. Amongst coaches and parents, high levels of trust and coping were reported; when asked if another parent or assistant coach helps run training or a game if they are unable to attend, 90% of coaches agreed. Further, 86% of parents agreed that they trust another parent/coach to react appropriately if their child suffers an injury during the game and they are absent.

### 3.6. Regression Results

Two binary regression analyses were conducted to characterize the perception of risk around asthma in organized youth team sports (coach sample; parent sample). Coefficients are presented as odds ratios (ORs) with 95% confidence intervals (CIs) (significance with *p* < 0.1 are also reported as our models intend to be explanatory and not predictive). When reference categories are reported, odds ratios can be interpreted as the odds of a respondent to rate asthma risk as high relative to the reference category, while controlling for other variables in the model. The models (Table 2 [Coaches] and Table 3 [Parents]) achieved Nagelkerke R square values of 0.727 (coaches) and 0.738 (parents), with 92.6% (coaches) and 86.7% (parents) of cases correctly classified. Significant associations (*p* < 0.1) are reported below.

#### 3.6.1. Physical Environment

Amongst parents specifically, those participating in indoor (OR: 0.001, 95% CI: (0.001, 0.36), *p* < 0.05) or outdoor (OR: 0.08, 95% CI: (0.01, 1.33), *p* < 0.1) physical environments were less likely to perceive asthma risk as high compared with participants of sport in both contexts.

#### 3.6.2. Environmental Health Risk

##### Risk Characteristics; Unknown

In the parent model, the Asthma Symptom Trigger score emerged as significant. For each increase by one in the score, the likelihood that a respondent would rate the risk of asthma as high decreased (OR: 0.59, 95% CI: (0.31, 1.1), *p* < 0.1).

##### Risk Characteristics; Exposure

Direct exposure was a significant predictor amongst coaches only, as those who were not personally affected by allergic disease were less likely to rate asthma risk as high (OR: 0.04, 95% CI: (0.002, 0.79), *p* < 0.05). With respect to indirect exposure, coaches who had other family members affected were less likely to perceive asthma risk as high (OR: 0.02, 95% CI: (0.001, 1.24), *p* < 0.1). Amongst parents, those who did not have a child impacted by three or more allergies were less likely to rate asthma risk as high (OR: 0.02, 95% CI: (0.001, 0.67), *p* < 0.05).

##### Mediators of Expectation

The Propensity for Risk score was a significant predictor in both the coach and parent models. For each increase by one in the score, the likelihood that a respondent would rate the risks of asthma as high increased (Coaches: OR: 1.32, 95% CI: (0.99, 1.77), *p* < 0.1; Parents: OR: 2.45, 95% CI: (1.37, 4.50), *p* < 0.01). Amongst coaches, the Environmental Attitudes score was a significant predictor of perceived asthma risk (OR: 1.94, CI: (0.92, 4.08), *p* < 0.1); this relationship was inverse amongst parents (OR: 0.49, CI: (0.23, 1.06), *p* < 0.1). 

Two trust and coping indicator variables were included in the coach and parent models; coach respondents who disagreed with the statement ‘If I am unable to attend/coach a practice, I will reschedule’, were less likely to rate asthma risk as high (OR: 0.1, 95% CI: (0.01, 1.22), *p* < 0.1). 

##### Socioeconomic Position and Demographics

Coaches of recreational sport (OR: 0.01, 95% CI: (0.001, 0.66), *p* < 0.05), and those with medical qualifications (OR:0.03, 95% CI: (0.001, 1.29), *p* < 0.1) were less likely to rank asthma risk as high. Gender was also a predictor amongst coaches; males were less likely to rate asthma risk as high (OR: 0.04, 95% CI: (0.001, 1.17), *p* < 0.1), compared with females. Education was also significant in the coach model; those with higher education (postsecondary: OR: 0.001, 95% CI (0.001, 1.22), *p* < 0.1; above postsecondary: OR: 0.017, 95% CI (0.001, 2.28), *p* < 0.1) were less likely to perceive asthma risk as high. 

## 4. Discussion

This paper applied Harrington and Elliott’s place-based conceptual framework [4] for understanding the public experience of risk to investigate the factors shaping asthma risk perception and document asthma risk perception outcomes amongst team sport coaches and parents of allergic athletes in Ontario. Multivariate results reveal that amongst coaches, those directly affected by allergic disease perceived asthma risk as higher than those not diagnosed. The relationship between exposure and risk perception is complex and direct experience can either enhance or mitigate perceived risk. For example, Whitmarsh found that flood victims are less likely to perceive potential effects of climate change as catastrophic compared with non-flood victims [35], while Mayer et al. report that direct exposure does not strongly predict risk perception of air pollution and climate change [36]. While coach direct exposure is associated with perceived risk, the relationship was inverse for indirect exposure. Further, the relationship with direct exposure did not exist amongst parents; all parent respondents are indirectly exposed to allergic disease, and a larger proportion are directly impacted themselves (46% of parents, 27% of coaches). Parent respondents may possess increased asthma management knowledge, and may have increased familiarity with asthma risk. Parents whose child was diagnosed with 3+ allergies were more likely to rank asthma risk as high. This is consistent with perceptions of food allergy, as Harrington et al. reported participants who had multiple allergies in the home were more likely to rank food allergy risk as high [13]. 

Amongst parents, trigger knowledge was negatively associated with risk perception. Prior knowledge is known to influence perceived risk [37], however the relationship is inconsistent. Sjoberg and Drottz-Sjoberg found that knowledge was negatively associated with nuclear risk perception [38], while Harrington et al. reported that those who received food allergy information prior to participation were more likely to rank food allergy risk as high [13]. The variance between knowledge and risk perception is consistent here; while trigger knowledge is associated with lower perceived risk, symptom knowledge is not related to risk perception amongst coaches or parents. Parents with high trigger knowledge may be more likely to sustainably manage their child’s asthma, and therefore perceive its risk as reduced.

Education was also significantly associated with asthma risk perception. Education is a significant predictor of perceived risk in other work, as those with higher levels of education generally perceive risk as lower [39]. This is consistent with respect to education status in the coach model. While completion of coach education was excluded from the multivariate models (at the time of data collection, no Canadian coaching education focused on environment and health/allergic disease), coaches with medical training were less likely to perceive asthma risk as high. This is unsurprising, as allergy-related emergencies are covered in standard first aid (e.g., EpiPen guidelines [40,41]), potentially increasing familiarity with allergic disease management amongst coaches.

Interestingly, one trust and coping indicator was associated with perceived risk. This relationship was inverse and existed only amongst coaches; those who disagreed that they would reschedule a practice when unable to attend were less likely to rank risk perception as high. While the relationship between risk perception and trust varies by context, trust measure, and type of risk [42], an inverse relationship between trust and risk perception is consistent with other work [43]. Amongst coaches, higher levels of trust may increase feelings of social support and reduce perceived vulnerability with respect to environmental health risks.

Finally, gender was also significantly associated with perceived risk amongst coaches, and males were less likely to perceive asthma risk as high. This is frequently observed in risk perception research [4,31,44], whereby white males are more likely to perceive risks to be low [44]. While reasons for the gender discrepancy remain unclear, in the context of health risks in sport, female coaches may socially construct (asthma) risk differently to males, due to the social construction of females as primary caregivers [13], or the reported propensity for females to consult physicians more frequently for health-related symptoms [45].

Following Harrington et al. and Krewski et al.’s [13,31,32] previous work exploring perceived health risks in Canada, asthma is ranked as the seventh highest risk (by both coaches and parents) amongst 17 health hazards. The majority of coaches (n = 60) and parents (n = 52) rank asthma as a moderate risk, and both coach and parent results demonstrate that asthma falls below similar health hazards, including other respiratory allergy and food allergy. While other forms of allergic disease, and hazards related to, or determinants of respiratory allergy and asthma appear in previous work (e.g., cigarette smoking, outdoor air quality [13,31,32], climate change, indoor air quality [13]), this survey was the first to include asthma and respiratory allergy in the list of health risks to Canadians. 

Limitations of this research exist. First, splitting coach and parent responses to conduct separate multivariate analyses reduced the sample size of both surveys. While this allowed for the inclusion of additional variables (e.g., questions only included in one survey), it may not be representative of the coach or asthmatic parent populations in Ontario. Similarly, while this research contributed to increasing our understanding of how coaches and parents in Ontario may perceive asthma risk, the coach and parent respondent samples were not geographically representative (e.g., the Regional Municipalities of Halton and Waterloo were most strongly represented), and significant relationships may exist beyond these results. Increasing the sample and ensuring representation from across Ontario (e.g., through broader recruitment) could increase reliability and reduce the variability of results. Next, the recruitment strategy could present a source of bias. Coaches were recruited to complete a survey related to the links between coaching, physical activity, the environment and health, while parents were recruited based on their child’s allergic disease. This could therefore impact how parents rank asthma/allergic disease. Thirdly, while this research aimed to understand subjective perceptions, risks with self-reported data exist; although results are anonymous, responses may in some cases differ from objective health assessments, questions may be misunderstood, and participants may respond how they perceive to be socially desirable. Finally, although various demographic backgrounds were represented, certain groups were under/over-represented, and analysis based on the region of residence was not possible due to small sample size and lack of geographical representation. Extending the survey to other stakeholders in sport could address this gap and allow for future analysis based on other environmental factors (e.g., physical or political environment based on the geographical region). 

## 5. Conclusions

While understanding perceptions of risk is essential for effective risk management, communication, and decision making [31], the ways in which youth team sport coaches and parents of allergic athletes in Ontario organized youth team sport perceive asthma in the context of sport participation are complex. Ensuring asthma is well-managed in a supportive youth team sport environment (e.g., involving coaches, teammates, parents, and sport providers) can contribute to how youth athletes impacted by asthma and respiratory allergy participate in, enjoy, and maintain physical activity and sport participation into adulthood. As policy-makers and sport governing bodies prioritize and manage the myriad of health risks that could impact athletes, considering the varied factors that contribute to perceived risk will be critical for effective risk communication, and to ultimately improve asthma management and governance in Ontario organized youth team sport.

## Figures and Tables

**Table 1:** Survey sample characteristics.

Demographic Variable		Number of Coach Respondents (%)	Number of Parent Respondents (%)
Gender	Male	52 (55%)	18 (20%)
	Female	41 (44%)	69 (77%)
	Did Not Identify	1 (1%)	3 (3%)
Age	20–29	24 (26%)	0 (0%)
	30–39	14 (15%)	17 (19%)
	40–49	43 (46%)	57 (63%)
	50–59	11 (12%)	13 (14%)
	60–69	2 (2%)	0 (0%)
	70+	0 (0%)	1 (1%)
	Did Not Identify	0 (0%)	2 (2%)
Education	Less than secondary school	0 (0%)	0 (0%)
	Secondary school	12 (13%)	8 (9%)
	Postsecondary (e.g., college or university bachelor level)	63 (67%)	60 (68%)
	Master’s/PhD/professional degree	19 (20%)	21 (23%)
	Did Not Identify	0 (0%)	0 (0%)
Immigrant Status	Canada Born	81 (86%)	73 (81%)
	Immigrant	13 (14%)	17 (19%)
Marital Status	Married	62 (66%)	73 (81%)
	Single	22 (23%)	0 (0%)
	Separated/Divorced	8 (9%)	13 (14%)
	Widowed	0 (0%)	3 (3%)
	Did Not Identify	2 (2%)	1 (1%)
Total		94 (100%)	90 (100%)

**Table 2:** Perceived asthma risk—binary regression results for coach participants.

Component of Framework			Explanatory Variables	Adjusted Odds Ratio	95% Confidence Interval	Significance ^
Physical Environment			Sport Environment: Both (ref.)	1.00	-	-
			Sport Environment: Outdoor	N/S	-	-
			Sport Environment: Indoor	N/S	-	-
Environmental Health Risk	Risk Characteristics	Unknown	Asthma Symptom Knowledge Score	N/S	-	-
			Asthma Symptom Trigger Score	N/S	-	-
	Exposure	Direct	Personal Allergic Disease: Yes (ref.)	1.00	-	-
			Personal Allergic Disease: No	0.04	(0.002, 0.79)	**
		Indirect	Other Family Members Affected: No (ref.)	1.00	-	-
			Other Family Members Affected: Yes	0.02	(0.001, 1.24)	*
	Mediators of Expectation	General Environmental Risk Attitudes	Propensity for Risk Score	1.32	(0.99, 1.77)	*
			Environmental Attitudes Score	1.94	(0.92, 4.08)	*
		Measures of Trust/Coping	Contact information for team members and their families is available for them to keep in touch: Agree	N/S	-	-
			If I am unable to attend/coach a practice, I will reschedule: Disagree	0.10	(0.01, 1.22)	*
	Socioeconomic Position and Demographics	Sport-Related	Sport Level: Competitive (ref.)	1.00	-	-
			Sport Level: Recreational	0.01	(0.001, 0.66)	**
			Sport Level: Other	N/S	-	-
			Medical Qualifications: No (ref.)	1.00	-	-
			Medical Qualifications: Yes	0.03	(0.001, 1.29)	*
			Years of Experience: 0–5 (ref.)	1.00	-	-
			Years of Experience: 6–15	N/S	-	-
			Years of Experience: 16+	N/S	-	-
		General	Gender: Female (ref.)	1.00	-	-
			Gender: Male	0.04	(0.001, 1.17)	*
			Canadian Born: Yes (ref.)	1.00	-	-
			Canadian Born: No	N/S	-	-
			Marital Status: Partnered (ref.)	1.00	-	-
			Marital Status: Not Partnered	N/S	-	-
			Education: Secondary (ref.)	1.00	-	-
			Education: Postsecondary (e.g., Bachelors or equivalent)	0.001	(0.001, 1.22)	*
			Education: Above (e.g., Masters, Doctorate)	0.017	(0.001, 2.28)	*
			Employment Status: Employed (ref.)	1.00	-	-
			Employment Status: Other (e.g., unemployed, volunteer)	N/S	-	-
			Age	N/S	-	-

*** *p* < 0.01, ** *p* < 0.05, * *p* < 0.1.

**Table 3:** Perceived asthma risk—binary regression results for parent participants.

Component of Framework			Explanatory Variables	Adjusted Odds Ratio	95% Confidence Interval	Significance ^
Physical Environment			Sport Environment: Both (ref.)	1.00	-	-
			Sport Environment: Outdoor	0.08	(0.01, 1.33)	*
			Sport Environment: Indoor	0.001	(0.001, 0.36)	**
Environmental Health Risk	Risk Characteristics	Unknown	Asthma Symptom Knowledge Score	N/S	-	-
			Asthma Trigger Knowledge Score	0.59	(0.31, 1.1)	*
	Exposure	Direct	Personal Allergic Disease: Yes (ref.)	1.00	-	-
			Personal Allergic Disease: No	N/S	-	-
		Indirect	Child with Asthma: Yes (ref.)	1.00	-	-
			Child with Asthma: No	N/S	-	-
			Child with Other Allergy: Yes (ref.)	1.00	-	-
			Child with Other Allergy: No	N/S	-	-
			Child with 3+ Allergies: Yes (ref.)	1.00	-	-
			Child with 3+ Allergies: No	0.02	(0.001, 0.67)	**
	Mediators of Expectation	General Environmental Risk Attitudes	Propensity for Risk Score	2.45	(1.37, 4.50)	***
			Environmental Attitudes Score	0.49	(0.23, 1.06)	*
		Measures of Trust/Coping	Contact information for team members and their families is available for them to keep in touch: Agree	N/S	-	-
			If I am unable to attend a practice or game, my child will usually not attend either: Disagree	N/S	-	-
	Socioeconomic Position and Demographics	Sport-Related	Sport Level: Competitive (ref.)	1.00	-	-
			Sport Level: Recreational	N/S	-	-
			Sport Level: Other	N/S	-	-
		General	Gender: Female (ref.)	1.00	-	-
			Gender: Male	N/S	-	-
			Canadian Born: Yes (ref.)	1.00	-	-
			Canadian Born: No	N/S	-	-
			Marital Status: Partnered (ref.)	1.00	-	-
			Marital Status: Not Partnered	N/S	-	-
			Education: Secondary (ref.)	1.00	-	-
			Education: Postsecondary (e.g., Bachelors or equivalent)	N/S	-	-
			Education: Above (e.g., Masters, Doctorate)	N/S	-	-
			Employment Status: Employed (ref.)	1.00	-	-
			Employment Status: Other (e.g., unemployed, volunteer)	N/S	-	-
			Age	N/S	-	-

*** *p* < 0.01, ** *p* < 0.05, * *p* < 0.1.
